# Acknowledgment to Reviewers of *Medical Sciences* in 2020

**DOI:** 10.3390/medsci9010004

**Published:** 2021-01-25

**Authors:** Medical Sciences Editorial Office

**Affiliations:** MDPI AG, St. Alban-Anlage 66, 4052 Basel, Switzerland

Peer review is the driving force of journal development, and reviewers are gatekeepers who ensure that *Medical Sciences* maintains its standards for the high quality of its published papers. Thanks to the cooperation of our reviewers, in 2020, the median time to first decision was 27 days and the median time to publication was 60.5 days. The editors would like to express their sincere gratitude to the following reviewers for their precious time and dedication, regardless of whether the papers were finally published:
Adams, Harold P.Mascia, Maria LidiaAlbert, Daniel ArthurMatrone, AntonioAlexander, H. DenisMeloni, BrunoAljohan, MohammedMercer, Derry KAlsaied, TarekMherekumombe, Martha F.Anwer, M. SawkatMinami, MasaakiArciniega, Alejandra BenitezMiranda, Joana P.Azzolini, ClaudioMiyamoto, ToshinobuBarnard, Ross T.Montomoli, CristinaBarros-Dios, Juan MiguelMuhammad, NaoshadBatule, Bhagwan SahebraoNapoli, ChristianBauer, StefanieNiemczyk, MariuszBlum, Jason L.Noll, MatiasBorger, JessicaNylander, KarinBrown, Joshua DavidOmatsu, TsutomuCantalupo, AnnaPaoletti, Anna MariaCapelli, IreneParuccini, NicolettaCarbone, JavierRadin, RachelCaria, PaolaRaggi, CarlaChaigne, AgatheRein, DietrichChang, Kwo-PingRewley, JeffreyChellan, NireshniRia, FrancescoChen, Wei-YuRiley, MalcolmChiechio, SantinaRipellino, PaoloChinegwundoh, FrancisRiza, ElenaChong, Poh HengRo, HyunjuChung, EricRogobete, Alexandru FlorinContreras, Rubén G.Rybka, JakubDachs, GabiSabbatinelli, JacopoDaina, EricaSahoo, SubhransuDelitala, AlessandroSahu, Ravi PDimitriadis, StavrosSammarco, GiuseppeDoi, KentSarbini, ShahrulDunn, Michael F.Sato, EiichiEcke, ThorstenSawall, StefanEmrich, Fabian C.Sciarrillo, Christina M.Enthoven, PaulSethi, GautamEvanson, Nathan K.Sevcikova, SabinaEvengård, BirgittaShabunin, Anton S.Ferguson, Sue A.Shankland, RebeccaFerrell, Jessica M.Sharafutdinov, IrshadFranklin, Renty B.Silvia, RattiFrimodt-Møller, NielsSironen, Anu I.Gao, ShuaiSkrzypek, KlaudiaGarcía-Barrado, María JoséȘlencu, Bogdan GabrielGąsior-Perczak, DanutaSmith, Webb AGera, RuchiSo, Wi-YoungGianesini, SergioSoRelle, Jeffrey AGnat, SebastianSteel, AmieGolebiowski, TomaszStella, AlessandroGordon, Catherine A.Stork, Christian J.Gori, TommasoSubramanian, ArulHanna, RamySuchkov, SergyHarrison-Bernard, LisaSueta, DaisukeHenning, MarcusSur, SubhayanHorvatits, ThomasSvahn, Tony MartinHsieh, Shu-ChenTarantini, StefanoHsu, Chung Y.Thumfart, JuliaHu, HuiTinnirello, AndreaJanssen, Patrick W. M.Van Gijn, Marielle EJones, LeahVenkatesan, ThiagarajanKadambi, Pradeep V.Vergne, MatthewKang, Kyung PyoVerhoeven, AdrieKang, Sang SunViggiano, DavideKlar, AgnesWang, ChengmingKouji, HaradaWang, Jie (Jane)Krieg, SandroWang, LiyeKurpisz, MaciejWang, MengyuKyriacou, AngelosWatanabe, EiichiLamas, AlexandreWax, MarkLavender, AndrewWesselius, AnkeLe Clec’h, WinkaWeström, BjörnLeff, ToddWoldeamanuel, Yohannes WoubishetLiu, Kejian (Jim)Wright, DavidLonardo, AmedeoXu, JianLu, QuanlongYamaki, JasonMagalhães, Fernando HenriqueYoung, Jennifer M.Maguire, DonoghZanini, FilippoManeta, EleniZhou, MinMantovani, AlessandroZhu, YuMartin-Loeches, IgnacioZuhl, Micah

